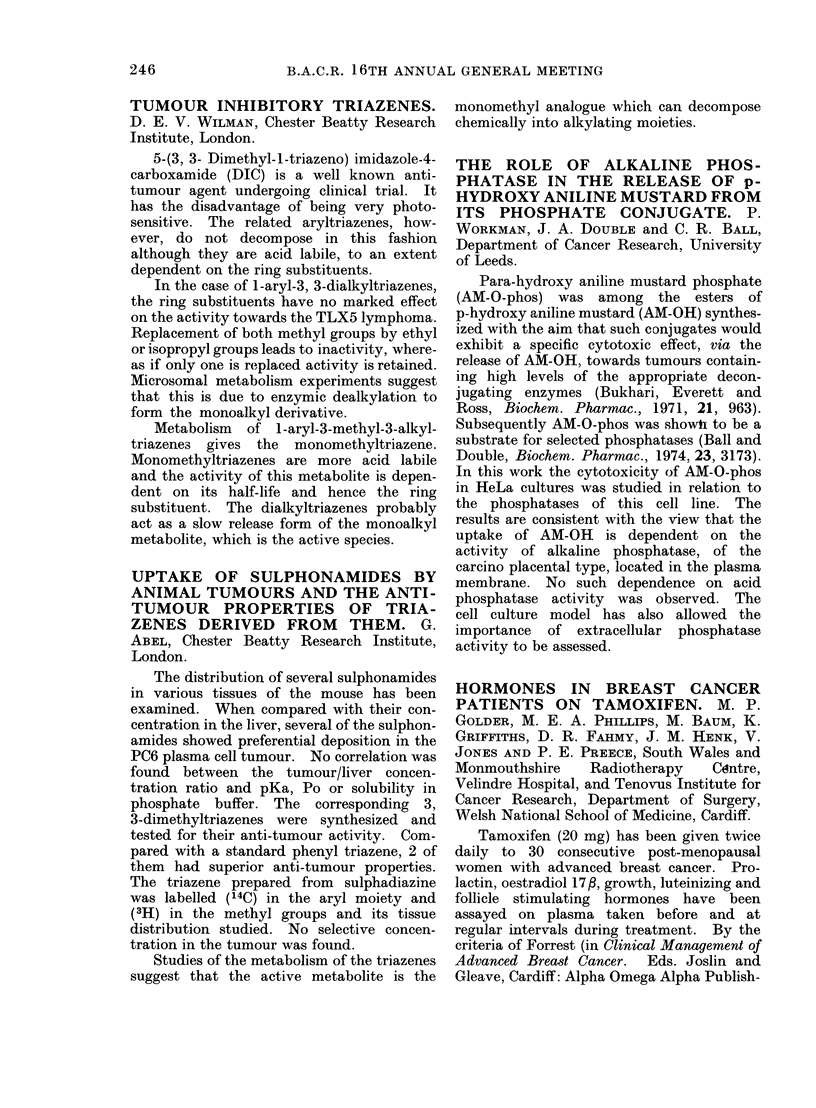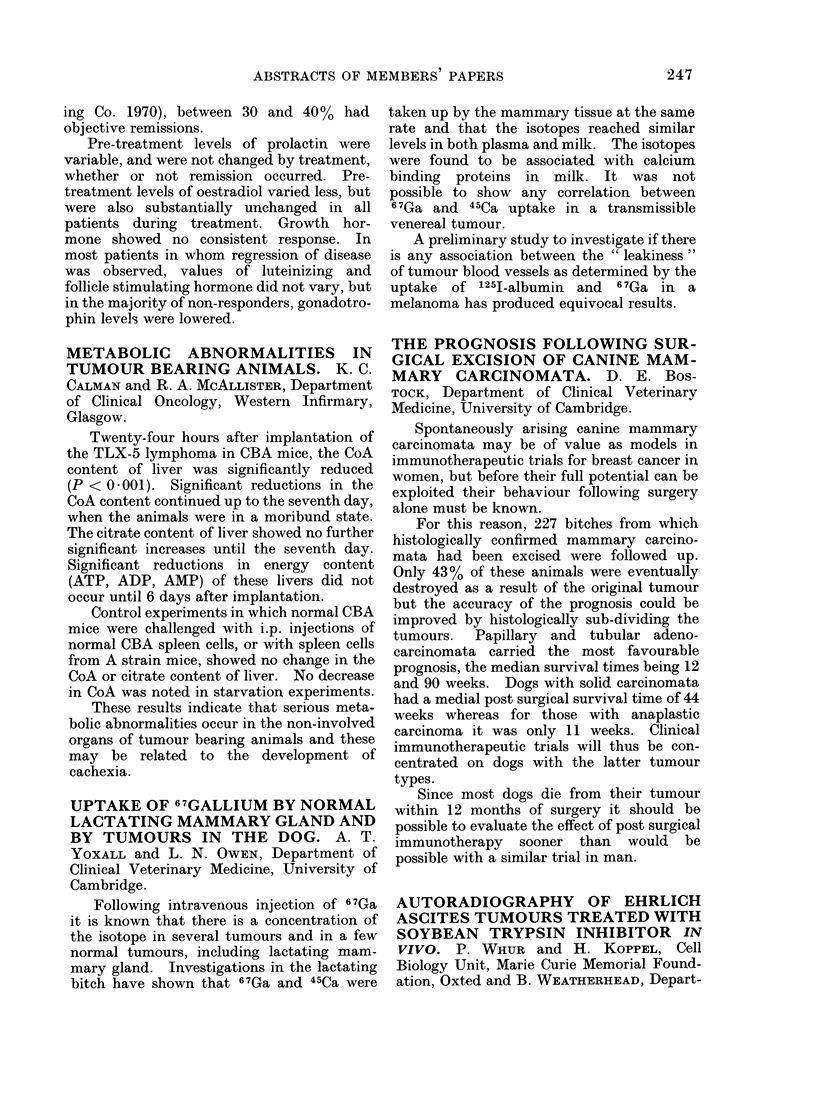# Proceedings: Hormones in breast cancer patients on tamoxifen.

**DOI:** 10.1038/bjc.1975.176

**Published:** 1975-08

**Authors:** M. P. Golder, M. E. Phillips, M. Baum, K. Griffiths, D. R. Fahmy, J. M. Henk, V. Jones, P. E. Preece


**HORMONES IN BREAST CANCER PATIENTS ON TAMOXIFEN.** M. P. GOLDER, M. E. A. PHILLIPS, M. BAUM, K. GRIFFITHS, D. R. FAHMY, J. M. HENK, V. JONES AND P. E. PREECE, South Wales and Monmouthshire Radiotherapy Centre, Velindre Hospital, and Tenovus Institute for Cancer Research, Department of Surgery, Welsh National School of Medicine, Cardiff.

Tamoxifen (20 mg) has been given twice daily to 30 consecutive post-menopausal women with advanced breast cancer. Prolactin, oestradiol 17$\beta$, growth, luteinizing and follicle stimulating hormones have been assayed on plasma taken before and at regular intervals during treatment. By the criteria of Forrest (in *Clinical Management of Advanced Breast Cancer*. Eds. Joslin and Gleave, Cardiff: Alpha Omega Alpha Publishing Co. 1970), between 30 and 40% had objective remissions.

Pre-treatment levels of prolactin were variable, and were not changed by treatment, whether or not remission occurred. Pre-treatment levels of oestradiol varied less, but were also substantially unchanged in all patients during treatment. Growth hormone showed no consistent response. In most patients in whom regression of disease was observed, values of luteinizing and follicle stimulating hormone did not vary, but in the majority of non-responders, gonadotrophin levels were lowered.